# Tumor Growth in Overdrive: Detailing an Aggressive Course of Hepatocellular Carcinoma

**DOI:** 10.1155/2024/4950398

**Published:** 2024-06-15

**Authors:** Simardeep Singh, Thilini Delungahawatta, Marcos Wolff, Christopher J. Haas

**Affiliations:** ^1^MedStar Union Memorial Hospital, Baltimore, MD, USA; ^2^MedStar Franklin Square Medical Center, Baltimore, MD, USA

## Abstract

Hepatocellular carcinoma ranks as the third leading cause of cancer-related mortality globally. We present a case of a rapidly progressive hepatocellular carcinoma in an 81-year-old female with metabolic abnormalities. The patient initially presented with non-specific signs and symptoms and was managed for sepsis of suspected urinary source. Unresolving laboratory markers led to repeat abdominal imaging demonstrating new hepatic lesions within six days. Biopsy confirmed moderately differentiated hepatocellular carcinoma. The patient received conservative inpatient treatment with recommendation for nutritional and performance status optimization prior to oncologic therapies, however continued to decline and passed away three months later.

## 1. Introduction

Hepatocellular carcinoma (HCC) is the 3^rd^ leading cause of cancer-related deaths worldwide, accounting for about 800,000 deaths annually [1]. It is a primary liver malignancy commonly linked with chronic liver tissue injury [2]. In one global qualitative study, over 50 percent of HCC cases were attributed to chronic hepatitis B virus (HBV) and 20 percent of cases were attributed to chronic hepatitis C virus (HCV) infection [3]. Other risk factors include environmental toxins [4], lifestyle factors such as excessive consumption of alcohol [5], genetic susceptibility [6, 7], and metabolic syndrome [8]. The latter is an umbrella term for metabolic disorders including obesity, type II diabetes, dyslipidemia, and elevated blood pressure [8].

While patients with metabolic syndrome are well known to be at risk of certain cardiovascular diseases, new data have also shown a correlation to the development of various types of cancers [9]. Indeed, disruption of several molecular signaling cascades commonly observed in subjects with metabolic syndrome has been implicated in hepatic carcinogenesis [8]. Albeit heterogeneity in tumor growth patterns [10], the burden of primary liver cancer is expected to rise over the next two decades [1], challenging current screening guidelines. Given that treatment options are highly dependent on tumor stage and underlying liver function [11], individualized screening timelines may be warranted.

Herein, we discuss a rare case of accelerated progression of HCC in a patient with only known metabolic abnormalities.

## 2. Case Presentation

An 81-year-old female with history of hypertension, hyperlipidemia, cerebrovascular accident, and coronary artery disease with multiple stenting and prior myocardial infarction presented to the emergency room with generalized malaise. The patient noted a 3-day history of cough with yellow sputum and diffuse abdominal pain with nausea, vomiting, poor appetite, and unspecified weight loss. Review of systems was further notable for subjective fevers, progressive weakness, and confusion. She denied any changes to her bowel movements or any urinary symptoms. No history of hepatitis infections or excessive use of alcohol or tobacco was noted.

On initial assessment, vitals were remarkable for hypoxia (SpO_2_ 87% (reference range: 93–100%)) and tachypnea (31 BR/min (reference range: 12–20 BR/min)); otherwise, the patient was afebrile, non-tachycardic, and normotensive. Physical exam was notable for lethargy, diminished mentation (alert and oriented to person and place only), mild generalized jaundice, scattered crackles to auscultation of the lungs bilaterally, and mild, non-focal tenderness to palpation of abdomen without rebound/guarding, organomegaly, or any masses. Laboratory diagnostics (Table 1) demonstrated mild anemia (hemoglobin, 9.9 g/dl (reference range: 12.5–16.5 g/dl)), thrombocytopenia (131 × 109/L (reference range: 145–400 × 109/L)), and leukocytosis (18.2 k/*μ*L (reference range: 4–10.8 k/*μ*L)). Coagulation panel showed an elevated PT (17.6 seconds (11.8–14.6 seconds)) and INR (1.4 (0.8–1.2)). Furthermore, liver function tests revealed an elevated total bilirubin (2.7 mg/dL (reference range: 0.2–0.9 mg/dL)) that was predominantly direct (2.10 mg/dL (reference range: 0.00–0.3 mg/dL)), aspartate aminotransferase (AST; 858 U/L (reference range: 0–33 U/L)), alanine aminotransferase (ALT; 473 U/L (reference range: 10–49 U/L)), and alkaline phosphatase (ALP; 475 U/L (reference range: 46–116 U/L)). Additionally, the patient had an acute kidney injury (creatinine 2.70 (reference range: 0.52–1.04 mg/dL)) and elevated lactic acid (8.9 mmol/L (reference range: 0.7–2 mmol/L)).

**Figure 1:**
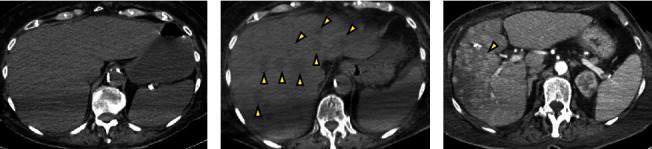
Computed tomography imaging of the abdomen and pelvis without intravenous contrast showing transverse view of (a) homogeneous appearing liver on day 1 with subsequent development of (b) ill-defined hypodense lesions (arrows) with the largest lesion measuring 5.6 cm within the central liver on day 6 and (c) repeat imaging with IV contrast showing progression of disease with innumerable masses at 2 months.

**Figure 2:**
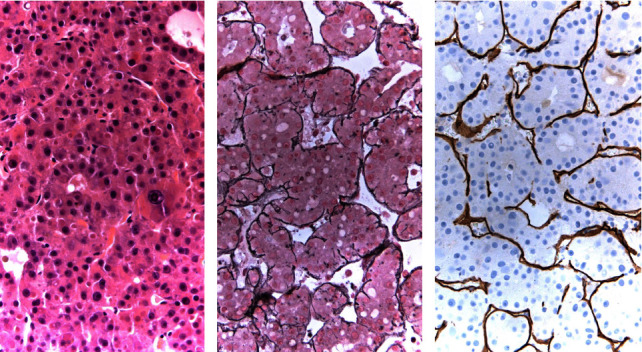
Histopathological examination of the right liver biopsy shows: (a) polygonal cells with eosinophilic cytoplasm, nuclear atypia, a high nuclear-to-cytoplasmic ratio, and prominent nucleoli (hematoxylin-eosin stain, ×40 magnification); (b) hepatocyte plate thickening (reticulin stain, ×40 magnification); and (c) strong and diffuse sinusoidal staining pattern (CD34 immunohistochemical stain, ×40 magnification). Collectively, these findings are consistent with a diagnosis of HCC.

The patient was started on broad-spectrum antibiotics (vancomycin and piperacillin-tazobactam), intravenous fluids, and supportive oxygen and admitted to the intermediate care unit for further evaluation and management of suspected sepsis. Given the kidney injury, a non-contrast CT of the chest, abdomen, and pelvis was performed. The test revealed a right proximal 4 mm urolithiasis with no significant hydronephrosis but prominent perinephric stranding and lungs with early markers of interstitial pneumonia. Subsequently, her blood cultures grew pan-sensitive *E. coli*. Sepsis was therefore thought to be secondary to a complicated UTI. The patient received nephrostomy tube placement and was continued on IV antibiotics. However, on day 6, she continued to complain of right-sided abdominal pain and had increasing leukocytosis (27.27 k/*μ*L (reference range: 4–10.8 k/*μ*L)). Infectious disease team was further consulted, and the patient underwent a repeat non-contrast CT scan of the abdomen and pelvis which then showed mildly enlarged liver measuring up to 21.3 cm with multiple ill-defined hypodense lesions within the liver which may reflect metastatic disease versus underlying primary malignancy (Figure 1(b)). Alpha-fetoprotein was ordered and resulted as less than 2.2 ng/mL (reference range: 0–8.0 ng/mL). The patient underwent USG biopsy of the liver with pathology confirming tumor comprised of polygonal cells (Figure 2). Further evaluation with non-contrast CT scan of the head, contrast-enhanced CT of the chest, abdomen, and pelvis, and bone scan did not show any metastatic disease. Abdominal pain was management with IV morphine with subsequent weaning to oral medications.

Oncology team was then consulted and treatment with immunotherapy was discussed; however, the patient had required optimization of nutritional and performance status prior to initiation. The patient was then discharged in a stable condition to subacute rehab with recommendation for outpatient follow-up with medical oncology. Unfortunately, the patient underwent multiple subsequent hospitalizations for recurrent malignant pleural effusions and developed a deep vein thrombosis, necessitating anticoagulation therapy which was further complicated by severe anemia. Due to her condition, she was not a suitable candidate for oncologic therapies, resulting in the progression of hepatic lesions that quickly replaced most of the normal liver parenchyma (Figure 1(c)). The patient became bedridden and elected for hospice care, passing away three months after the initial diagnosis.

## 3. Discussion

About 70–90% of primary liver cancers worldwide are HCC, known to be the fastest growing cause of cancer-related death with advanced cases carrying a 5-year survival rate of less than 20% [2, 12, 13]. While viral hepatitis and excessive alcohol use are commonly identified risk factors for HCC, a significant subset of patients (5% to 30%) have no predisposing factors [8]. We have described a case of rapidly progressing HCC in a patient with hypertension, hyperlipidemia, and associated vascular complications. Recent research has linked the increased risk and poor outcomes of several types of cancer with metabolic syndrome and its various components [14]. For instance, in one meta-analysis examining a correlation between type II diabetes and hepatic carcinogenesis, a 2.5-fold increase in the risk for HCC was noted [15]. Similarly, another meta-analysis showed that in addition to impaired fasting glucose, dyslipoproteinemia, hypertension, and obesity were each significantly associated with the development of HCC (*p* < 0.0001) [16]. Furthermore, in HCC patients with metabolic syndrome as the only identifiable risk factor, distinct histopathological features have been described including the absence of significant fibrosis [17]. These results suggest specific molecular pathways of liver tumorigenesis in such individuals. Indeed, the heightened production of oxidative stress and reactive oxygen species inducing cancer promoting mutations, high levels of insulin growth factor-1 increasing cell turnover and inhibiting apoptosis, and dysregulated inflammatory cytokine responses have been shown to influence HCC development [8].

Due to the inherently low contrast resolution of the liver, the evaluation of hepatic lesions is ideally conducted with contrast enhancement during the portal venous phase [18]. However, in this case, two separate assessments performed without contrast and using the same CT machine had revealed HCC progression within 6 days. To our knowledge, this pattern of tumor growth is the most aggressive case described to date. The current HCC screening recommendation to perform abdominal ultrasonography every 6 months for high-risk individuals is largely based on accumulated estimates of tumor volume doubling time of 70–120 days from previous cases [10]. Specifically, in a meta-analysis of 20 studies, 35% of HCC patients had rapid tumor development (TVDT <3 months), 27.4% had intermediate growth (TVDT 3–9 months), and 37.6% had indolent growth (TVDT >9 months). Growth patterns varied depending on where the investigations were conducted; studies in Asia showed a higher percentage of individuals with aggressive tumors (43.8% versus 25.5%, respectively, *p* < 0.001). Three investigations also reported correlation between individuals with persistent HBV infection and shorter TVDT. The authors, however, could not draw any correlation between the degree of liver dysfunction, such as use of the Child–Pugh score, and growth patterns or patient demographics, such as age or sex. Several other studies have suggested a linear relationship between smaller tumor diameter and shorter TVDT (i.e., more rapid growth) [19–22]. Higher alpha-fetoprotein levels have also been linked to rapid HCC progression; however, these findings were contradictory to findings of other studies that did not establish a link [19, 23–25]. More recently, molecular genetic studies have suggested that alterations in components of the spliceosome complex, particularly overexpression of proteins PRPF8 and SF3B1, may lead to dysregulation of gene expression favoring rapid HCC progression [26, 27]. Additional evidence from case series and experimental data is required to enhance our understanding of aggressive HCC tumorigenesis and its implications for screening.

## 4. Conclusion

The rapid progression of hepatocellular carcinoma is observed in the presented case, particularly within a remarkably short 6-day timeframe. This case highlights how current surveillance guidelines may miss lesions in patients with more aggressive tumor growth patterns. However, further research is needed to propose tailored surveillance protocols, given the present heterogeneity in tumor growth rates.

## Figures and Tables

**Table 1:** Laboratory diagnostics of an 81-year-old female presenting with abdominal pain.

Lab	Value	Reference range
Hemoglobin	9.9 g/dl	12.5–16.5 g/dl
Leukocytes	18.2 k/*μ*L	4-10.8 k/*μ*L
Platelets	131 × 10^9^/L	145–400 × 10^9^/L
PT	17.6 seconds	11.8–14.6 seconds
INR	1.4	0.8–1.2
Total bilirubin	2.7 mg/dL	0.2–0.9 mg/dL
Direct bilirubin	2.10 mg/dL	0.00–0.3 mg/dL
AST	858 U/L	0–33 U/L
ALT	473 U/L	10–49 U/L
ALP	475 U/L	46–116 U/L
Creatinine	2.7 mg/dL	0.52–1.04 mg/dL
Lactic acid	8.9 mmol/L	0.7–2 mmol/L

## Data Availability

All data used to support the findings of this case report are available as part of the article and cited references.
